# Mechanisms and roles of mitochondrial localisation and dynamics in neuronal function

**DOI:** 10.1042/NS20200008

**Published:** 2020-06-01

**Authors:** Richard Seager, Laura Lee, Jeremy M. Henley, Kevin A. Wilkinson

**Affiliations:** School of Biochemistry, Centre for Synaptic Plasticity, Biomedical Sciences Building, University of Bristol, University Walk, Bristol BS8 1TD, U.K.

**Keywords:** DRP1, fission, fusion, mitochondria, mitochondrial dynamics, post translational modification

## Abstract

Neurons are highly polarised, complex and incredibly energy intensive cells, and their demand for ATP during neuronal transmission is primarily met by oxidative phosphorylation by mitochondria. Thus, maintaining the health and efficient function of mitochondria is vital for neuronal integrity, viability and synaptic activity. Mitochondria do not exist in isolation, but constantly undergo cycles of fusion and fission, and are actively transported around the neuron to sites of high energy demand. Intriguingly, axonal and dendritic mitochondria exhibit different morphologies. In axons mitochondria are small and sparse whereas in dendrites they are larger and more densely packed. The transport mechanisms and mitochondrial dynamics that underlie these differences, and their functional implications, have been the focus of concerted investigation. Moreover, it is now clear that deficiencies in mitochondrial dynamics can be a primary factor in many neurodegenerative diseases. Here, we review the role that mitochondrial dynamics play in neuronal function, how these processes support synaptic transmission and how mitochondrial dysfunction is implicated in neurodegenerative disease.

## Introduction

Our understanding of mitochondria has changed dramatically over the past few decades. Utilising improved imaging techniques, it is now appreciated that mitochondria do not exist as isolated structures (often how they are depicted in textbooks), rather they comprise an interconnected, cell-wide, dynamic network. Indeed, the word *mitochondria* derives from the Greek words *mitos*, meaning thread, and *chondros*, meaning grain, alluding to the many morphologies the mitochondria adopt, recognised over 100 years ago (Benda, cited in [1]).

The continuous fusion, fission (division) and movement of mitochondria, together with other essential behaviours, including cristae remodelling, biogenesis and mitophagy (selective mitochondrial autophagy) are collectively termed ‘mitochondrial dynamics’. Together, these processes fine-tune mitochondrial function to match energy supply and demand, to facilitate transport to sites of high energy expenditure, to allow exchange of components between mitochondria and to mediate the specific removal of damaged organelles. Fusion and fission are governed by the interplay between different GTPases, which form fusion and fission machinery at the mitochondrial membranes. The balance between these opposing processes are stringently regulated and can be adjusted to modify the overall mitochondrial morphology in response to changing energy demands and cellular stress [2,3].

Furthermore, the concept of mitochondria as simply the ‘powerhouse of the cell’ has expanded. It is now clear that beyond ATP generation via oxidative phosphorylation (OXPHOS), mitochondria are integral to many signalling pathways. Mitochondria act as signalling hubs, buffer Ca^2+^, have roles in apoptosis, and are sites of reactive oxygen species (ROS) signalling and of metabolite generation (reviewed in [4–6]).

## Mitochondria in neurons

Neurons are morphologically complex, excitable and polarised cells with immense energy demands [7–9]. Mitochondria are directed to sites of energy demand and are integral to neuronal function [10]. However, the details of how spatiotemporal regulation of mitochondria contribute to synaptic function have remained elusive.

**Figure 1:**
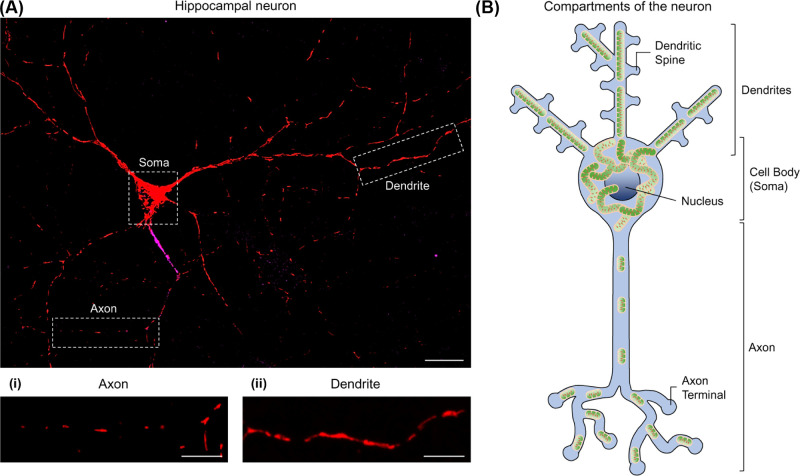
Neuronal mitochondria exhibit compartment-specific morphologies (**A**) Primary rat hippocampal neuron expressing a mitochondrially targeted fluorescent protein (MitoDS-Red). Ankyrin-G staining (magenta) shows the axonal initial segment, used to identify the axon. Highlighted within the boxes are the axonal (i) and dendritic (ii) compartments (enlarged beneath); scale bar: 20 µm, 10 µm in enlargements. (**B**) Schematic showing the compartments of a neuron, depicting the long axon and multiple dendrites from the cell body (soma), containing the nucleus. Axonal mitochondria are small and sparse, whereas dendritic mitochondria are larger and occupy a greater volume of the process. Mitochondria are densely packed within the soma.

Intriguingly, mitochondria exhibit varied morphologies between the axonal and dendritic compartments. Axonal mitochondria are small and sparsely distributed, whereas dendritic mitochondria appear elongated and occupy a greater volume of the neurite (see Figure 1) [11,12]. Moreover, axonal and dendritic mitochondria differ in their movement, metabolism and responses to neuronal activity [11,13–15], with axonal mitochondria exhibiting more dynamic behaviour than dendritic mitochondria [16].

These differences presumably arise from differential regulation of mitochondrial dynamics in response to compartment-specific processes. Because of their importance in maintaining neuronal integrity and function, the mechanisms and significance of these differences are of great interest [12,16–19]. Here, we outline current knowledge of how mitochondrial dynamics impacts on their transport, localisation, Ca^2+^ buffering and energy provision and highlight the central role of mitochondrial dynamics in neuronal function and dysfunction.

## Neuronal biology imposes an energetic burden

Neurons require large amounts of ATP to perform a wide range of energy intensive functions. Indeed, the human brain constitutes ∼2% of body mass, yet utilises ∼20% of the O_2_ consumed by the body [20,21], underscoring the intensive energy demands of neuronal activity. Maintaining resting membrane potential and restoring ionic balance after depolarisation are high energy expenditure processes [7–9], with synaptic vesicle recycling representing a major ATP demand during activity [22,23]. Additionally, the extensive and complex architecture of neurons imposes a geometric challenge for neuronal cell biology, such as the transport of proteins and organelles from the soma to distal sites, raising the question of how energy demands are met at distal sites (reviewed in [24,25]).

For example, some peripheral axons extend distances of >1 m, and subtypes of neurons (e.g. dopaminergic neurons in the substantia nigra), have remarkably extensive axonal arbours with ∼4.5 m of total axonal length and 1–2.4 million synapses [26]. Furthermore, some calculations estimate >100 m total axonal length in basal forebrain cholinergic neurons in humans [27]. Indeed, it has been estimated that ∼7.1 × 10^8^ ATP molecules are required for a single neuron to undergo, and recover from, one action potential [20]. How is this huge energy demand fulfilled?

Glycolysis was classically thought to supply most neuronal ATP [28,29]. Investigation of the relationship between presynaptic ATP supply and demand using an optical reporter of ATP showed that in resting cells (using TTX to inhibit action potentials) ATP levels remained relatively stable [23]. The fact that ATP levels are not significantly altered by inhibition of activity suggests that either spontaneous activity does not impose an appreciable energetic burden on neurons or ATP required for synaptic activity is rapidly synthesised ‘on demand’ [23]. By examining the rate of ATP depletion following blockade of ATP synthesis by glycolysis and OXPHOS, it was demonstrated that glycolysis supports neuronal ATP levels in the absence of activity, whereas OXPHOS inhibition had no effect [23]. Analogous experiments conducted during stimulation revealed that both glycolysis and OXPHOS are required to supply ATP during activity. Together, these findings show that neuronal activity drives ATP generation [23], confirming previous reports that OXPHOS is the major source of energy during neuronal activity and synaptic transmission to support spine growth, cytoskeletal rearrangements and protein synthesis [13,16,30].

**Figure 2:**
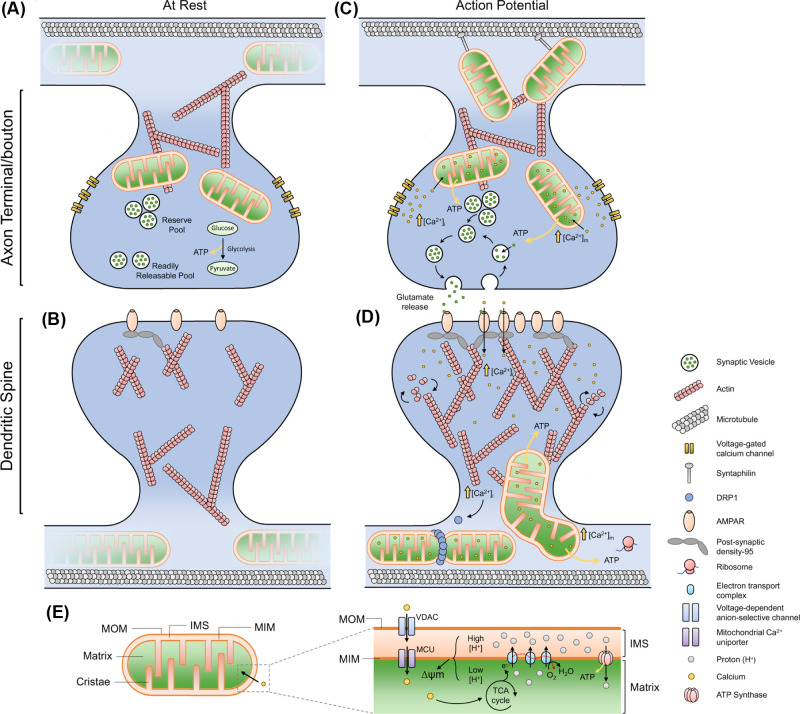
Roles of mitochondrial dynamics and calcium in support of neuronal activity and plasticity (**A**) Axonal mitochondria are very dynamic, exhibiting both anterograde and retrograde movement. At the presynapse, glycolysis and OXPHOS support basal activity of the synapse. (**B**) Dendritic mitochondria are larger than axonal mitochondria and exhibit more stabilised behaviour, with a small number of spines containing mitochondria. (**C**) During an action potential, an influx of Ca^2+^ rapidly increases cytoplasmic [Ca^2+^], also increasing mitochondria [Ca^2+^], which stimulates OXPHOS activity and increased ATP generation. The enhanced ATP generation powers energy demanding processes such as vesicle recycling, endocytosis and mobilisation of the reserve pool. Local mitochondria stop trafficking and are stabilised by the action of syntaphilin (anchoring protein) and rearrangements of the Miro-TRAK complex. Axonal mitochondria also buffer Ca^2+^ to prevent sustained elevations of [Ca^2+^]_i_, thus regulating continuous rounds of firing, while also preventing asynchronous transmission. (**D**) Ca^2+^ influx at the postsynapse causes dendritic mitochondria to protrude into the spine, and also causes mitochondrial fission, which regulates Ca^2+^ transients in the mitochondria during activity. Dendritic mitochondria provide ATP for actin-dynamics, supporting growth of the postsynaptic density, surface expression of neurotransmitter receptors and supports local translation. (**E**) Schematic of mitochondrial compartments and Ca^2+^ influx. Mitochondria consist of two membranes: the mitochondrial outer membrane (MOM) and the mitochondrial inner membrane (MIM). The region formed between the MOM and MIM is called the intermembrane space (IMS). The MIM has extensive folds, which form the cristae, increasing the surface area of the membrane which houses the electron transport chain (ETC) components. Substrates from the tricarboxylic acid (TCA) cycle act as electron donors to the ETC, which couples electron transfer with the shuttling of protons across the MIM into the IMS, forming a higher concentration of protons in the IMS compared to the matrix. This generates a concentration gradient and an electrical potential (due to charge separation), termed the mitochondrial membrane potential (∆ψm). The ∆ψm is the driving force for ATP synthesis, as protons pass down their concentration and electrical gradient, passing through the enzyme ATP Synthase, forming ATP. ∆ψm is also a driving force for Ca^2+^ sequestration in the matrix. Ca^2+^ must traverse both the MOM and MIM to enter the matrix. The voltage-dependent anion-selective channel (VDAC) and the mitochondrial Ca^2+^ uniporter (MCU) make the MOM and MIM permeable to Ca^2+^, respectively. Matrix localised Ca^2+^ stimulates the TCA cycle and enhances mitochondrial OXPHOS function.

Increasing synaptic activity decreases the motion of dendritic mitochondria [31] and enhances their localisation at synapses [11]. Correspondingly, during postsynaptic depolarisation, dendritic mitochondria become clustered at the base of spines and protrude into them [13], whereas decreasing network activity increases mitochondrial length, their occupancy along the dendrites and elevates the number of mobile mitochondria [11]. In the axon, increasing activity enhances the localisation of mitochondria at synapses [15] and decreases mitochondrial length [11]. Suppression of synaptic activity leads to a reduction in the number of presynapses with mitochondria [15] and a decrease in the number of mobile mitochondria [11], while others have found that decreasing activity increases axonal mitochondrial mobility [32], although this discrepancy is likely due to neuronal maturity, with older cultures being more sensitive to network activity suppression [32]. These findings demonstrate that synaptic activity has profound effects on mitochondrial behaviour and localisation (Figure 2).

## Calcium homeostasis

One major role of synaptic mitochondria is to buffer intracellular Ca^2+^ [33–35]. Briefly, during an action potential, voltage-gated Ca^2+^ channels open to allow an influx of Ca^2+^ into the presynaptic terminal, ultimately leading to neurotransmitter release [36]. The activity of the electron transport chain (ETC) pumping protons out of the matrix to generate the mitochondrial membrane potential (∆ψm) favours sequestration of cations (i.e. Ca^2+^) within the matrix (see Figure 2E, reviewed [33–35]). Thus, mitochondria have a large capacity to store and buffer intracellular Ca^2+^, which modulates neurotransmission [37–40].

### Regulation of presynaptic Ca^2+^ by mitochondria

To enter the mitochondrial matrix, Ca^2+^ must pass through two channels; the voltage-dependent anion-selective channel (VDAC) and the mitochondrial Ca^2+^ uniporter (MCU) (Figure 2E) [41]. During rest, mitochondrial ([Ca^2+^]_m_) and cytosolic ([Ca^2+^]_i_) Ca^2+^ concentrations are similar at ∼100 nM, but neuronal depolarisation causes presynaptic [Ca^2+^]_i_ to rise sharply by ∼10–20-fold, and local mitochondria sequester considerable amounts of this Ca^2+^ [37,42]. The presynaptic spike in [Ca^2+^]_i_ is followed by an exponential decay back to resting levels. Inhibition of mitochondrial Ca^2+^ influx extends this decay time, resulting in sustained elevations of presynaptic [Ca^2+^]_i_ [37,42]. Indeed, mitochondrial Ca^2+^ buffering capacity is pivotal for presynaptic Ca^2+^ flux and regulating synaptic strength and neurotransmitter release [37–40]. No difference in presynaptic [Ca^2+^]_i_ was detected between presynapses containing or lacking mitochondria under resting conditions in cultured hippocampal neurons [15]. However, upon continuous depolarisation, synapses lacking mitochondria had enhanced [Ca^2+^]_i_ and vesicle release [15]. These findings broadly agree with an earlier study in the *Drosophila* neuromuscular junction (NMJ), although this study did note ∼2-fold increase in resting [Ca^2+^]_i_ at sites without mitochondria [43], suggesting mitochondria play a role in basal Ca^2+^ buffering in some neuronal types.

Mitochondria destabilisation causes asynchronous synaptic vesicle release during depolarisation, compared with more stable release at synapses with local mitochondria [44], which is dependent on mitochondrial function [45]. These studies establish a direct link between mitochondrial positioning and synaptic transmission, but is there a relationship between mitochondrial morphology and Ca^2+^ buffering capacity?

A recent study demonstrated that enhancing fusion increases both the rate and the maximum capacity of Ca^2+^ taken up by the mitochondria, whereas the opposite occurs in fragmented mitochondria [46]. The authors speculate this is due to the greater matrix volume of more fused mitochondria compared with the relatively smaller volume of fragmented mitochondria [46], although it should be noted that mitochondrial size impacts on ∆ψm [17,47], which is a driving force for Ca^2+^ influx.

### Regulation of Ca^2+^ by postsynaptic mitochondria

The role of dendritic mitochondria and Ca^2+^ in regulating plasticity has received less attention. A recent report showed that LTP causes local dendritic mitochondria to undergo extensive fission, and that mitochondria exhibit Ca^2+^ elevations during LTP [19]. Postsynaptic mitochondrial fission and increased matrix Ca^2+^ is dependent on depolarisation and Ca^2+^ influx [19], agreeing with an earlier study which demonstrated that depolarisation induces rapid fission of mitochondria [48]. Decreasing dendritic mitochondrial size by promoting fission decreases postsynaptic Ca^2+^ fluctuations during activity, resulting in reduced protein synthesis in stimulated spines [16], demonstrating dendritic mitochondrial size influences plasticity.

### Effects of Ca^2+^ sequestration on mitochondrial function

Early work demonstrated that Ca^2+^ enhances the activity of a number of mitochondrial enzymes involved in the tricarboxylic acid cycle [49–51]. Subsequently, it was shown that elevations in [Ca^2+^]_i_ increase ATP generation in mitochondria [52]. This report identified three key findings: (i) the concentration of ATP is proportional to the rise in [Ca^2+^]_m_; (ii) ATP generation is dependent on the availability of oxidative substrates; and (iii) mitochondria are ‘primed’ by [Ca^2+^]_m_ elevations, since [Ca^2+^]_m_ elevation does not greatly increase ATP when oxidative substrates are absent. However, once the stimulus is removed and oxidative substrates replenished, there is a large increase in ATP generation, thus, mitochondria can match ATP generation to energy demand [52].

In summary, these studies indicate that mitochondrial dynamics directly affect neurotransmission and plasticity at both sides of the synapse (Figure 2). An emerging concept is that large mitochondria can sequester more Ca^2+^, but clearly Ca^2+^ handling is important in both axons and dendrites, so the significance of the larger mitochondria in dendrites versus the smaller axonal mitochondria requires further investigation.

## Mitochondrial transport

Although a level of mitochondrial biogenesis can occur in the axon [53] the majority occurs in the soma [54]. Thus, mitochondrial transport is essential to supply distal sites as and when needed. Depending on the experimental model, approximately 10–40% of neuronal mitochondria are mobile at any one time [24]. Their dynamic behaviours include anterograde and retrograde movement, switching direction, pausing briefly, docking, fusing and dividing [55].

### Miro and TRAK proteins

**Figure 3:**
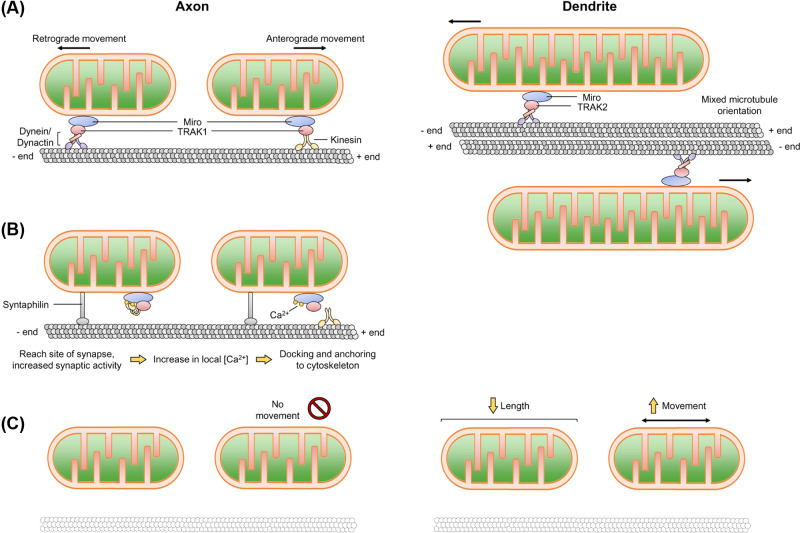
Mitochondrial transport in axons and dendrites (**A**) Mitochondria are transported in an anterograde and retrograde fashion along microtubules via kinesin and dynein/dynactin motor proteins. Microtubules are arranged from minus to plus-ends away from the cell body, whereas microtubules in the dendrite are arranged non-uniformly. The adaptor protein TRAK1 preferentially localises within axons, while TRAK2 preferentially localises to dendrites. (**B**) Upon an action potential or reaching a site of high energy demand characterised by an increase in [Ca^2+^]_i_, a reversible rearrangement of the motor and adaptor complex occurs, causing axonal mitochondria to pause. There are a number of models of how this occurs (i) Miro binds to Ca^2+^ and induces a conformational change in kinesin, releasing it from the microtubule tract and (ii) Miro binding to Ca^2+^ causes it to dissociate from kinesin. Syntaphilin also helps to immobilise mitochondria to the microtubule. How/if dendritic mitochondria are stabilised in a similar manner remains to be established. (**C**) Disruption of the neuronal cytoskeleton (actin and microtubules) stabilises axonal mitochondria, whereas dendritic mitochondria become shorter and more dynamic.

Trafficking and docking behaviour is mediated by the mitochondrial outer membrane (MOM) proteins Miro and the trafficking kinesin proteins (TRAK), which act as adaptors linking the molecular motors kinesin and dynein/dynactin to Miro [25,56,57]. There are two Miro proteins (Miro1/2), and two TRAK proteins (TRAK1/2). TRAK1 can bind both kinesin and the dynein/dynactin complex, while TRAK2 favours association with dynein/dynactin. In general, TRAK1 is broadly axonally localised and TRAK2 primarily mediates dendritic trafficking [58]. Microtubules in the axon are uniformly organised with the plus-ends arranged away from the soma, whereas the dendritic microtubules exhibit a mixed arrangement [59]. This results in kinesins driving anterograde transport and dyneins facilitating retrograde transport in axons, while dynein is the primary motor protein in dendrite transport [58,60,61] (see Figure 3A, neuronal transport extensively reviewed in [24,25,62–66]).

Miro1-knockout (Miro1^−/−^) mice die shortly after birth, but Miro2^−/−^ mice are viable [67,68]. These differences possibly reflect different roles in development [69], although Miro1 can compensate for loss of Miro2, indicating a level of redundancy [69]. Hippocampal Miro1^−/−^ neurons exhibit fewer mobile mitochondria, in both axons and dendrites, whereas Miro2-knockout has no effect [67]. The trafficking impairment in Miro1^−/−^ neurons causes mitochondria to accumulate in the proximal dendrites and be almost absent in distal dendrites, resulting in reduced total dendritic length. This redistribution of mitochondria also correlates with increased proximal, and reduced distal, dendritic branching [67]. Similarly, Miro1-knockout in mouse forebrain led to a progressive reduction of distal dendritic branching and shortening over time, confirming the importance for appropriate mitochondrial trafficking and distribution in mature neurons [67]. Since mitochondria in axons are similarly distributed in control and Miro1^−/−^ cells *in vitro*, and no effects were observed *in vivo* [67], Miro1 plays less of a role in the axon. Intriguingly, however, conflicting evidence suggests roles for Miro1 in motor neuron development and axonal branching *in vivo*, and that it also regulates axonal mitochondrial mobility in cultured cortical neurons [68].

As for Miro, knockdown of TRAK proteins reduces mitochondrial mobility. TRAK1-knockdown in hippocampal neurons results in fewer mobile mitochondria in the axon [58,70], but the velocity and proportion of anterograde and retrograde movements is unchanged [71]. Impaired mitochondrial distribution due to loss of TRAK1 correlates with reduced axonal branching and length [58]. Similar transport defects have been reported in cortical neurons [71], although these reports also note a reduction in the number of mobile dendritic mitochondria [58,71]. TRAK2-knockdown reduces the number of mobile dendritic mitochondria [58,71], leading to fewer and shorter dendrites in hippocampal neurons [58] and Purkinje cells [30]. Remarkably, the defect in dendritic length in Purkinje cells upon TRAK2-knockdown is rescued by supplementation of creatine (which enhances mitochondrial function and ATP production) [30], demonstrating that failure in mitochondrial transport restricts energy supply in neurites.

TRAK2-knockdown has been reported to have no effect in axons [30,70]; however, another study showed that TRAK2-deficiency reduces the number of mobile mitochondria in both compartments in cortical and hippocampal neurons. The greatest effects were observed in younger neurons [71], possibly reflecting developmental changes in mitochondrial mobility and expression of TRAK proteins during development [71]. Indeed, mitochondria in cultured cortical neurons become less mobile as neurons mature, in both the axon and dendrites, whereas this is only observed in the axons of hippocampal neurons. TRAK2 levels decrease during development, compared with the relatively stable expression of TRAK1 [71]. The discrepancy in the observations noted above may therefore reflect the different ages and neuronal type used to conduct the experiments.

Together, these studies demonstrate that appropriate mitochondrial distribution regulates proper morphological development and is essential for neurite integrity in mature neurons by supplying adequate amounts of ATP.

## Mitochondrial positioning

Two signals regulate the targeting and docking of mitochondria, namely the local ATP/ADP ratio and [Ca^2+^]_i_. Areas of ATP depletion, or conversely increases in ADP levels, such as at active synapses, cause mitochondria to slow their movement [72]. Increased [Ca^2+^] can reversibly rearrange the Miro-TRAK-kinesin complex, transiently pausing mitochondria where Ca^2+^ concentrations are greatest [31,73,74]. Multiple models of how Ca^2+^ mediates this rearrangement have been proposed (Figure 3B, reviewed in [10,74,75]).

### Mitochondria at the presynapse

Syntaphilin is a mitochondria-associated protein specifically expressed in the axon, which supports mitochondrial docking by binding to microtubule tracks, in effect anchoring mitochondria to the cytoskeleton (Figure 3B) [76]. Loss of syntaphilin increases mitochondrial mobility and distribution, resulting in a reduction of mitochondrial localisation at presynapses and elevated [Ca^2+^]_i_ following high-intensity stimulation [76]. Syntaphilin-deficient neurons exhibited no significant change in basal synaptic transmission [76]. Others have reported syntaphilin-knockout neurons have greater variability in excitatory postsynaptic currents (EPSCs), whereas overexpressing syntaphilin decreases mitochondrial mobility with a concomitant decrease in EPSC variability [44]. Moreover, mitochondria passing-by, or moving away from synapses, increases the variability in EPSCs and reduces successive synaptic transmission [44]. Indeed, ATP supplied by local mitochondria is responsible for continuous and stable vesicle release, whereas ATP levels are depleted and do not recover in the absence of mitochondria [44]. These results agree with an earlier study showing that synaptic mitochondria supply adequate ATP levels for continuous synaptic transmission and mobilisation of the reserve pool of vesicles [43]. These findings highlight the close relationship between mitochondrial transport, synaptic localisation and neurotransmission.

Presynaptic terminals and dendritic spines are enriched in actin filaments [77], and the actin-based motor myosin also plays a role in mitochondrial transport, possibly by disengaging mitochondria from microtubule-based transport [66]. A recent study identified that simultaneously disrupting the actin and microtubule cytoskeleton causes differential effects in axons and dendrites – axonal mitochondria became stabilised, whereas dendritic mitochondria became shorter and more dynamic [16]. These findings highlight potentially differential compartment-specific mitochondrial–cytoskeletal interactions, and further investigation is required into the possible different requirements/roles of the cytoskeleton in axons and dendrites (see Figure 3C).

### Mitochondria at the postsynapse

In contrast with the presynapse where there are substantial numbers of mitochondria under basal conditions [44], it has been reported that only ∼10% of dendritic spines contain mitochondria at rest [13]. Blockade of action potentials with TTX increases mitochondrial movement, whereas postsynaptic depolarisation decreases movement [11,13]. Moreover, synaptic activity recruits mitochondria to the postsynaptic density [13]. Local depletion of dendritic mitochondria had no effect on protein synthesis, suggesting ambient ATP levels are sufficient under basal conditions [16]. However, upon stimulation, spines depleted of mitochondria had impaired spine morphology changes and protein synthesis, which could be partially restored by expression of creatine kinase, which enhances ATP production [16]. These activity recruited, spine-associated mitochondria support new protein synthesis and spine growth [13,16] and also cytoskeletal rearrangements [30] by supplying sufficient ATP for these energy demanding processes.

Together, these studies indicate that there is a reciprocal arrangement in which synaptic activity regulates the number of local, stationary mitochondria, and that the proximity of stationary mitochondria directly supports synaptic activity and strength. See Figure 2 for roles of axonal and dendritic mitochondria during rest and synaptic activity, which has been extensively reviewed [10,73,74,78].

## Regulation of the mitochondrial network

In addition to referring to fusion, fission and transport, mitochondrial dynamics also encompasses broader behaviours, such as mitophagy, cristae remodelling, endoplasmic reticulum–mitochondrial contact sites and biogenesis [2,79,80]. The dynamic nature of the mitochondrial network confers a high degree of sensitivity and adaptability to changing metabolic demands, synaptic activity, and cellular stress. The highly orchestrated mechanisms of fusion, fission and transport adapt mitochondrial morphology and motility to restore homeostasis, and to remove damage to maintain a healthy, functional mitochondrial population [81–83].

The overall shape of the mitochondrial network is regulated by the two opposing forces of fusion and fission. Under basal conditions, mitochondria constantly undergo cycles of fusion and fission in equilibrium, with no net change in morphology [84,85]. However, the balance can be tipped to favour one process or the other. Increasing fusion, or decreasing fission, will result in a more connected network. Conversely, increasing fission, or inhibiting fusion, will result in a more fragmented mitochondrial network.

Fusion and fission are mediated by GTPases which utilise GTP hydrolysis to induce membrane fusion or scission. These processes have been extensively reviewed [3,79,80], but here we briefly discuss the mechanisms of the fusion/fission machinery and their biological consequences.

## Mitochondrial fusion

Mitochondrial fusion is conserved in all eukaryotic cells, from yeast to humans [86], and is essential for life, since disruption of mitochondrial fusion is embryonically lethal [87,88]. Fusion maintains a biochemically and functionally homogeneous population of mitochondria, achieved by ‘rescuing’ mitochondria that may be functioning at a sub-optimal level by complementing mitochondrial components, in effect ‘diluting’ damage [81]. Increased fusion during stress, known as stress-induced mitochondrial hyperfusion (SIMH), prevents mitophagy, maintains OXPHOS and preserves mitochondrial integrity [89]. Furthermore, SIMH protects cells from apoptosis and maintains ATP levels during starvation stress [90]. Correspondingly, fusion-deficient cells have reduced growth, wide-spread differences in ∆ψm and reduced respiration [47]. Thus, an emerging concept is that fusion of the mitochondrial network is cytoprotective, particularly during stress [90,91], and that loss of fusion predisposes cells to apoptosis [92,93], indicating that the fusion/fission balance affects sensitivity to apoptotic stimuli [93]. Pioneering work demonstrated that fusion occurs between two partner mitochondria that have sufficient ∆ψm, whereas those that are depolarised are fusion-deficient [84]. In this way, only biochemically functional mitochondria can fuse back with the network, while dysfunctional mitochondria are isolated and degraded.

Mitochondrial outer membrane (MOM) fusion is mediated by the mitofusin proteins Mfn1 and Mfn2, which form homo and heterodimers between the two opposing membranes [94]. Mfn-overexpression leads to mitochondrial elongation [95–97], and Mfn1/2-null cells exhibit severe mitochondrial fragmentation, but Mfn1 can rescue fusion deficient Mfn2-null cells, and *vice versa*, suggesting a level of redundancy [88]. However, Mfn1 GTPase activity is higher than that of Mfn2 [98], Mfn2 acts as a tether between the mitochondria and endoplasmic reticulum [99] and Mfn1 is required for OPA1-mediated fusion [97], demonstrating the existence of separate, non-overlapping functions between the Mfn proteins.

Mitochondrial inner membrane (MIM) fusion is regulated by optic atrophy protein 1 (OPA1). OPA1-depletion induces mitochondrial fragmentation [97,100]. OPA1 also has roles in shaping the mitochondrial cristae junctions, which house the majority of ATP synthase, complex III and cytochrome *c*. OPA1-knockdown triggers mitochondrial fragmentation, loss of ∆ψm and apoptosis [92,101], demonstrating its roles in regulating OXPHOS and apoptosis [102].

## Regulating the fusion machinery by post-translational modification

### Mitofusins

Mfn1 accumulates following short treatments with the mitochondrial complex III inhibitor antimycin A, resulting in SIMH. Prolonged treatment induces Mfn1 ubiquitination by the mitochondrial E3 ubiquitin ligase MARCH5, an interaction that involves Mfn1 acetylation [103]. Glucose starvation, which increases the bioenergetic demand on mitochondrial metabolism, leads to deacetylation by HDAC6 [104]. This presumably impairs the interaction between Mfn1 and MARCH5 and prevents ubiquitination and degradation, thus promoting fusion. Indeed, Mfn1-null cells are sensitised to stress-induced cell death [103]. Mfn1 phosphorylation by the mitogen-activated protein kinase kinase (MEK)/Extracellular-signal-regulated kinase (ERK) pathway induces mitochondrial fragmentation and promotes apoptosis [105], indicating that alterations in the levels of Mfn1, and its modifications, may determine cellular outcomes in response to stress.

An interaction has also been noted between MARCH5 and Mfn2 [106], although MARCH5-knockdown has no effect on Mfn2 protein levels [103]. Other reports show that depolarisation of mitochondria using CCCP (which dissipates ∆ψm and prevents OXPHOS) causes Mfn1 and Mfn2 ubiquitination by the E3 ligase parkin. This leads to proteasomal degradation of the fusion proteins and prevents re-fusion of dysfunctional mitochondria [107,108]. It has also been shown that Mfn2 is phosphorylated by the kinase PINK1, which is a prerequisite for recruitment of the ubiquitin ligase parkin and depolarisation-induced mitophagy [109].

Ubiquitin chains can form by multiple conjugation patterns, the most common being the K48-chain, leading to degradation, whereas atypical linkages can have non-degradative functions (reviewed in [110–112]). Interestingly, parkin conjugates atypical poly-ubiquitin chains to Mfn1, suggestive of non-degradative functions [113], although the significance of these modifications requires investigation.

### Optic atrophy protein 1

The MIM protein OPA1 is proteolytically cleaved at two sites by the mitochondrial membrane bound metalloproteases OMA1 and YME1L [114]. Differential cleavage generates different OPA1 isoforms (termed long and short OPA1 (L-OPA1, S-OPA1)) and the ratio between these isoforms determines the level of fusion and fission [114]. Although the mechanism is not fully understood, it is thought that OMA1 processing increases the S-OPA1/L-OPA1 ratio, which facilitates fission/prevents fusion during mitochondrial dysfunction [89]. YME1L activity is stimulated by efficient OXPHOS and may play a role in constitutive OPA1 activity [115,116], (reviewed in [89,114]).

## Mitochondrial fission

The major fission protein is the evolutionarily conserved mechanochemical GTPase dynamin-related protein 1 (DRP1), which constantly cycles between the cytoplasm and the MOM [117]. DRP1-null mice die at embryonic day ∼11.5, suggesting a role for DRP1 in early development [118,119]. At the MOM, DRP1 oligomerises and forms a helix around the mitochondria, and via GTP-hydrolysis constricts and induces membrane scission [120–122]. DRP1-knockout cells, or cells expressing a GTPase dead mutant, exhibit hyperfused mitochondria [119,121]. A recent *in vivo* study demonstrated that ablation of DRP1 in muscle results in altered mitochondrial morphology, decreased mitochondrial respiration and disrupted ETC complex assembly [123]. Similar defects in mitochondrial function were observed in a mouse model of DRP1-knockout in cardiomyocytes [124]. Preventing fission by knockdown of DRP1 in cultured HeLa cells leads to decreased ∆ψm, imparied ATP generation, loss of mtDNA and enhanced ROS [125]. These findings demonstrate that fission is required to maintain mitochondrial function and integrity. There is also evidence implicating another GTPase, dynamin-2, in mitochondrial fission [126], although this has been questioned [127,128].

DRP1 is recruited to the MOM by receptors, of which there are four in metazoans; mitochondrial fission protein 1 (Fis1), mitochondrial fission factor (MFF) and mitochondrial dynamics proteins of 49 and 51kDa (MiD49 and MiD51). Although the DRP1 receptors can function independently and have some level of redundancy, not all the DRP1 receptors are equal at promoting fission [129]. Systematic knockout of each DRP1 receptor demonstrates different capacities for DRP1 recruitment and different effects on morphology [129]. MFF has the greatest effect on morphology and DRP1 recruitment, whereas Fis1 has a minor role in fission [130,131]. The role of the MiD proteins was initially elusive, since exogenous expression of MiD51 induces mitochondrial elongation, but increases DRP1 association with mitochondria [132]. However, knockdown of both MiD proteins results in a fused network [132]. An emerging concept is that the MiD proteins sequester DRP1 on the MOM in an inactive state [131,133] by inhibiting DRP1 GTPase activity, while MFF enhances DRP1 GTPase activity [129].

Furthermore, knockout of the DRP1 receptors confers resistance to various stimuli such as fragmentation induced by the OXPHOS inhibitor CCCP, apoptosis and cytochrome *c* release, albeit to differing degrees of protection [129,131,134]. These findings emphasise the importance for DRP1 recruitment and mitochondrial fission under extreme cellular stress.

Fission is important to ensure equal distribution of mitochondria to daughter cells during cell division [135,136]. However, in postmitotic cells such as neurons, fission plays an important role in transport and distribution of mitochondria to distal neurites [12,13,43]. Fission is also important for quality control; damaged mitochondria are eliminated by mitophagy by the PINK1/parkin pathway [137–140], a process that involves mitochondrial fission [108,124]. Overexpression of either PINK1 or parkin fragments neuronal mitochondria in a DRP1-dependent manner [141]. This pathway also degrades the trafficking protein Miro [142] and Mfn proteins [108] to prevent mitochondrial mobility and potential re-fusion.

## Regulating the fission machinery by post-translational modification

### Dynamin-related protein 1

#### Phosphorylation

DRP1 is phosphorylated at two sites, S616 and S637, with seemingly differential effects on mitochondrial morphology. CDK1-mediated phosphorylation of DRP1 at S616 (pDRP1^S616^) during cell division promotes mitochondrial fission to ensure even distribution of mitochondria to the two daughter cells [136]. Phosphorylation at the S637 site (pDRP1^S637^) by PKA inhibits fission, protecting cells against mitophagy during nutrient deprivation and cell stress [90,143,144], whereas calcineurin-mediated dephosphorylation at DRP1^S637^ promotes translocation to the MOM and cell death [144,145]. Additionally, pDRP1^S637^ reduces the GTPase activity of DRP1 *in vitro* [143], suggesting that phosphorylation regulates both the localisation and activity of DRP1.

CDK5 is an atypical CDK that is highly expressed in postmitotic cells such as neurons, and has many non-cell cycle functions, including regulating synaptic transmission, synaptic vesicle cycling, neuronal migration and plasticity [146]. CDK5-mediated phosphorylation at DRP1^S616^ promotes fission during excitotoxic stress and neuronal cell death [147]. However, CDK5 phosphorylation of DRP1^S616^ was shown to increase as neurons mature, and corresponds to increasing mitochondrial length [148]. Clearly, further work will be required to determine how phosphorylation at the same site can lead to reportedly differential effects on mitochondrial morphology in neurons.

PKCδ-mediated phosphorylation of DRP1^S616^ has been reported to promote mitochondrial association and fragmentation under mitochondrial stress [149]. PKA-mediated phosphorylation at S637 elongates neuronal mitochondria [150], conferring neuroprotection against rotenone-induced cell death (inhibitor of ETC complex I) [151], whereas the phosphatase PP2A dephosphorylates pDRP1^S637^, resulting in fragmentation [150]. Calcineurin-mediated dephosphorylation of pDRP1^S637^ also promotes fission [144,145], and increasing the level of pDRP1^S637^ leads to mitochondrial elongation and confers neuroprotection against mitochondrial stress [151]. In apparent contrast, it has been reported that Ca^2+^ influx during neuronal activity induces CaMKIα-mediated phosphorylation of DRP1^S637^, which promotes its interaction with Fis1 and fission [48], while others have demonstrated that the Ca^2+^-CaMKII-pDRP1^S616^ axis promotes mitochondrial fission during LTP [19]. To address these seemingly contradictory observations, it will be necessary to further investigate the role of Ca^2+^ influx in regulating the DRP1 phosphorylation-state. Nonetheless, these reports highlight the link between synaptic activity and DRP1-mediated regulation of mitochondrial morphology.

#### Ubiquitination

The ubiquitin ligase MARCH5 has been reported to ubiquitinate DRP1 to control its degradation, leading to unopposed fusion [152]. However, MARCH5-null cells have also been shown to exhibit normal levels of DRP1 [103,153]. Further examination of these contradictory findings is required to better understand MARCH5-mediated ubiquitination of DRP1. The ubiquitin ligase parkin has also been reported to mediate DRP1 ubiquitination, leading to degradation [154].

#### SUMOylation

A modification similar to ubiquitination is the small ubiquitin-like modifier (SUMO), of which there are three major isoforms, SUMO1 and SUMO2/3, (reviewed in [155–157]). DRP1 has been reported to be modified by all three SUMO isoforms, with different outcomes for DRP1 function. SUMO1-ylated DRP1 is recruited to the mitochondria to promote fission [158,159], an effect which is antagonised by the deSUMOylating enzyme SENP5 [160]. SUMO1-modified DRP1 has roles in mitochondrial-ER contact site formation, and is required for Ca^2+^ transfer from the ER to mitochondria, cristae remodelling and cytochrome *c* release under apoptotic conditions [161]. On the other hand, SUMO2/3 modification of DRP1 partitions DRP1 in the cytosol during oxygen-glucose deprivation (OGD, an *in vitro* model of ischemia) [162], by reducing DRP1 binding to MFF [163]. However, upon termination of OGD and reperfusion, DRP1 is deSUMOylated by SENP3, allowing it to bind to MFF and initiate mitochondrial fragmentation and cell death [162]. Thus, the SUMOylation status of DRP1 represents an important determinant of cell fate following ischemic stress.

#### Other PTMs of dynamin-related protein 1

Other PTMs of DRP1 have been characterised, for example, DRP1 is modified by O-GlcNAcylation, which decreases pDRP1^S637^ levels in cardiomyocytes and leads to fragmentation [164]. Furthermore, modification by S-Nitrosylation increases mitochondrial fragmentation in neurons [165,166], and it was recently demonstrated that reduced S-Nitrosylation decreases pDRP1^S616^ levels, resulting in a mitochondrial elongation phenotype in neurons [167].

These studies demonstrate the plethora of signalling pathways that impinge on the PTM state of DRP1. How these relate and coordinate DRP1 function, and under what conditions, to regulate mitochondrial morphology and function remains to be explored further in neurons.

### Receptors of dynamin-related protein 1

Relatively little is known about the regulation of the DRP1 receptors, but recent reports have indicated a complex interplay between them.

#### Phosphorylation

MFF is phosphorylated by AMP-activated protein kinase (AMPK) at S155 and S172 [168–171]. AMPK is a major regulator of cellular energy homeostasis [172]. AMPK-mediated phosphorylation of MFF promotes mitochondrial fragmentation following treatment with OXPHOS inhibitors or AMPK activators [171]. Significantly, a phospho-mimetic mutant of MFF was sufficient to induce mitochondrial fragmentation to the same degree as observed by AMPK activation; however, a double phospho-null mutant failed to enhance DRP1 recruitment when treated with an AMPK activator or rotenone. Indeed, expression of MFF in cortical neurons *in vivo* resulted in a fragmented phenotype in the dendrites, which was not observed with a non-phosphorylatable MFF mutant. Thus, AMPK-mediated phosphorylation of MFF is an important determinant of neuronal mitochondrial morphology, and is necessary and sufficient to promote fission during bioenergetic stress [171].

#### Ubiquitination

Fis1 has been reported to be ubiquitinated by MARCH5, regulating its turnover [152]. MARCH5 also mediates ubiquitination and degradation of MiD49 upon mitochondrial stress and apoptosis stimuli [153], and MARCH5/MiD49 double-knockout cells exhibit little change in mitochondrial morphology, indicating MARCH5 function is MiD49-dependent [153]. However, while it has been reported that MARCH5-overexpression leads to mitochondrial elongation [106,152] and MARCH5^−/−^ cells exhibit increased fragmentation [153], another study reported MARCH5-knockdown increases fusion [173]. Clearly, further examination of the role of MARCH5 on mitochondrial morphology is required, and how MARCH5-mediated regulation of Mfn1, MiD49, Fis1 and DRP1 relate, and under what conditions they occur, remains to be explored.

Parkin-mediated MFF ubiquitination in response to mitochondrial depolarisation recruits the autophagy adaptor p62, which subsequently facilitates the clearance of damaged mitochondria [174]. Parkin is responsible for a significant amount of MFF ubiquitination, regulating its constitutive turnover [175]; however, there is evidence that at least one other ubiquitin ligase targets MFF [175]. Similar to Mfn1, MFF is also poly-ubiquitinated by atypical chains [175], indicative of a non-degradative function. However, the function of these atypical chains remains to be investigated.

### Complex interactions between DRP1 receptors

As mentioned, MiD proteins sequester inactive DRP1 on the MOM and can inhibit its GTPase activity [129,131,133]. A recent report offers an elegant mechanism as to how MiD proteins confer this inhibitory effect, whereby MiD proteins act as a ‘molecular bridge’ between DRP1 and MFF, forming a trimeric complex in which MiD promotes binding between DRP1 and MFF [176]. Too little MiD expression reduces DRP1–MFF binding, too much MiD outcompetes MFF for DRP1 binding [176]. This model offers an explanation to the observations that both overexpression and knockdown of MiD induces fusion [132,133]. How this complex is regulated offers an exciting area for future research.

Live-cell imaging has confirmed a high degree of co-localisation of MiD49, MiD51, DRP1 and MFF [177], and FRET and close proximity biotinylation experiments show a close association between MiD51, DRP1 and MFF [129]. In combination, these reports suggest that DRP1 adaptor proteins interact and cooperatively mediate fission. Indeed, Fis1 also binds to Mfn proteins and impairs the GTPase activity of the fusion machinery, suggesting that Fis1 may act as a negative regulator of fusion, rather than, or in addition to being, a positive regulator of fission [178].

**Figure 4:**
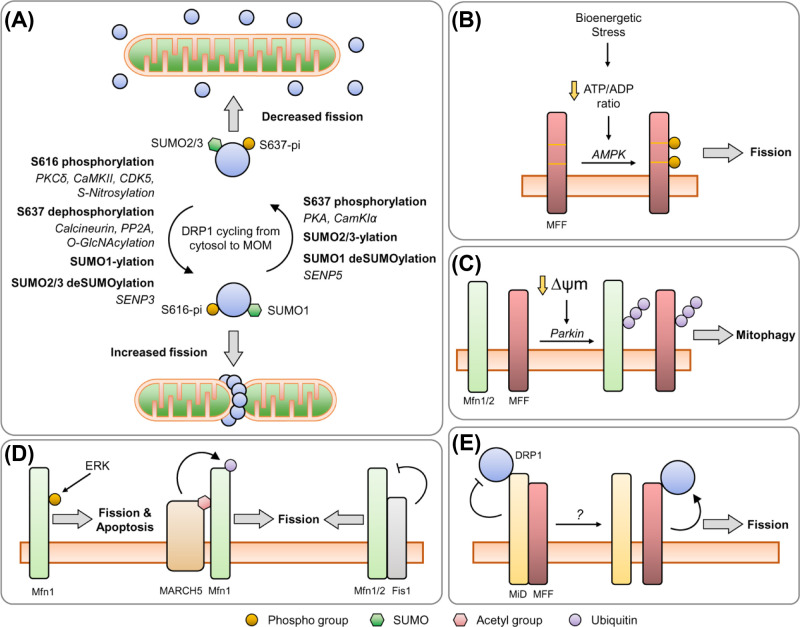
Regulation of mitochondrial dynamics proteins (**A**) DRP1 constantly cycles from the cytosol to the MOM. Phosphorylation at DRP1^S616^, mediated by PKCδ and CaMKII, promotes mitochondrial association and fission. Phosphorylation at DRP1^S637^ is mediated by PKA, which promotes mitochondrial elongation by inhibiting DRP1 GTPase activity and reducing MOM association, which is antagonised by the phosphatases calcineurin and PP2A. CDK5 has been demonstrated to phosphorylate DRP1^S616^, with reportedly contrasting effects on neuronal mitochondrial morphology, while CaMKIα-mediated phosphorylation at DRP1^S637^ has been shown to promote fission. Further research is required to confirm the roles of CDK5 and CaMKIα in DRP1 phosphorylation and function. S-Nitrosylation enhances pDRP1^S616^ levels, whereas O-GlcNAcylation reduces pDRP1^S637^ levels. Modification by SUMO1 promotes fission, whereas SUMO2/3 modified DRP1 sequesters DRP1 in the cytosol, preventing fission. The deSUMOylating enzymes SENP5 and SENP3 remove SUMO1 and SUMO2/3 from DRP1, respectively. (**B**) Following bioenergetic stress, leading to a reduction in the ATP/ADP ratio, AMPK is activated. AMPK phosphorylates MFF at S155 and S172, leading to fragmentation of the mitochondria. (**C**) Parkin activation in response to damaged mitochondria (i.e. dissipation of the ∆ψm) ubiquitinates many substrates on the MOM, leading to recruitment of the mitophagy machinery and degradation of the mitochondria. The two mitofusin proteins and MFF have been identified as parkin targets. Parkin-mediated ubiquitination of Mfn1/2 results in degradation, thus prevents fusion. Parkin-mediated ubiquitination of MFF recruits the mitophagy adaptor p62, leading to elimination of damaged mitochondria. (**D**) Under mitochondrial stress, MARCH5 ubiquitinates Mfn1, promoted by Mfn1 acetylation, which leads to Mfn1 degradation and fission. ERK-mediated phosphorylation of Mfn1 promotes mitochondrial fragmentation and apoptosis. Fis1 can bind to Mfn1 and inhibit its GTPase activity, thus promoting fission. (**E**) MiD proteins can impair the GTPase activity of DRP1, while MFF promotes DRP1 activity. DRP1-MiD-MFF exist as a trimeric complex, whereby MiD proteins facilitate binding between DRP1 and MFF; however, a mechanism of how this is regulated remains to be determined.

Overall, appropriately balanced fission and fusion are vital to sustain energy production, to maintain mitochondrial health, to remove damaged mitochondria and for the induction of apoptosis. Complex mechanisms, including PTMs, integrate the cell signalling pathways and stress response systems to regulate dynamic and context-specific alterations in mitochondrial morphology (see Figure 4 for an overview of the regulation of mitochondrial fusion/fission proteins. For reviews see [3,179,180]).

## Mitochondrial dynamics in axons

Studies that have manipulated fusion and fission proteins have advanced our understanding of the role these proteins play in the distribution and function of mitochondria within axons and dendrites.

### Effects of impairing fusion

#### Mfn proteins

A study using *Drosophila* larvae segmental nerves showed that knockdown of Marf (the fly ortholog of the Mfn proteins) induced mitochondrial fragmentation along the axon, accumulation within the soma and progressively fewer mitochondria from proximal to distal sections of the axon [17]. At the NMJ mitochondria were almost completely absent, and in the flight muscles of adult flies mitochondria showed internal ultrastructural abnormalities and derangement of cristae structure [17]. Fusion-deficient mitochondria also caused functional deficits in ATP generation, O_2_ consumption and decreased ∆ψm [17].

A study using a forebrain-specific inducible Mfn2-knockout mouse model reported swollen mitochondria with ultrastructural abnormalities in the soma and an almost complete lack of mitochondria in the axons [181]. Ablation of Mfn2 led to reduced ETC protein expression and progressive oxidative stress, inflammation and neuronal death [181]. These findings are consistent with an earlier *in vivo* study showing that loss of Mfn2 results in mitochondrial fragmentation, reduced distribution along the axon and degeneration in dopaminergic neurons [182]. Cultured Mfn2-null dopaminergic neurons exhibit reduced anterograde and retrograde transport with reduced velocity and enhanced time spent stationary [183], agreeing with findings made in the *Drosophila* larvae [17], implicating an additional role of Mfn2 in regulating transport [183].

Overexpression of Mfn1 in primary cortical neurons elongates mitochondria in the soma and along neurites [105]. Nonetheless, the role of Mfn1 in controlling compartment-specific mitochondrial morphology awaits further investigation.

#### OPA1

Loss of OPA1 in dopaminergic neurons causes a similar fragmentation phenotype as Mfn2-knockdown, but no observable transport defect [183]. This suggests that affecting fusion does not lead to a transport deficit *per se*. In contrast, and similar to the phenotype upon loss of Marf, OPA1 depletion in *Drosophila* larvae segmental neurons reduces mitochondrial mobility, increases pausing, impedes OXPHOS function and significantly reduces the number of mitochondria at the NMJ [17]. This study found that in both OPA1 or Marf knockdown neurons the density of mitochondria progressively decreased from proximal to distal sections of the axon, indicating a length-dependent defect in transport [17]. Detailed examination of mitochondrial behaviour upon OPA1 loss in *Drosophila* larvae shows that mitochondria have reduced fusion and fission events within the axons, enhanced retrograde movement, smaller stationary mitochondria compared to controls and reduced distribution into distal portions of the axon [184].

A study of cultured retinal ganglion cells also observed mitochondrial aggregation upon OPA1-knockdown [185], again implying that OPA1-mediated fusion is required for proper mitochondrial distribution. Moreover, OPA1 loss in primary rat cortical neurons is associated with a reduction in the presynaptic proteins synapsin and synaptophysin [186], indicating that fusion is required to support synaptogenesis and/or maintenance of synapses.

### Effects of impairing mitochondrial fission

#### DRP1

DRP1-knockout neural stem cells (NS-DRP1^−/−^ cells) display decreased protein levels of Mfn1/2 and increased OPA1 processing to the fusion-incompetent S-OPA1 isoform, suggesting a compensatory mechanism to reduce excessive fusion [118]. However, mitochondria aggregate within the soma with reduced trafficking into the neurites. NS-DRP1^−/−^ cells also exhibit decreased expression of the presynaptic marker synaptophysin and reduced neurite number [118]. These results suggest defective mitochondrial distribution impairs synaptic maintenance, agreeing with reports of OPA1 loss [186]. Similarly, a study in the *Drosophila* NMJ showed that DRP1-knockout flies have clustered mitochondria in the soma and reduced axonal mitochondria at synapses. However, no concomitant change in the number or size of synapses was detected [43]. The authors also report a failure in neurotransmitter release during high intensity stimulation and impaired mobilisation of the reserve pool of synaptic vesicles, which they attribute to a lack of ATP [43]. In hippocampal neurons from forebrain-specific DRP1-knockout mice, mitochondria cluster in the soma with reduced presynaptic mitochondria, but experience normal electrophysiological properties at rest and no change in the number of presynapses [187]. However, a reduction in ATP and O_2_ consumption levels were detected between DRP1-knockout and control hippocampal cells, and following intense stimulation, synaptic transmission was impaired [187], agreeing with other findings [43]. Interestingly, ATP levels in the soma of DRP-knockout and control hippocampal cells were similar, whereas ATP levels were not maintained in the axon of DRP1-knockout cells during activity, causing impaired synaptic vesicle recycling [188]. This study noted that loss of DRP1 increases the size of mitochondria along the axon but did not affect the number of synapses with mitochondria [188]. This suggests there is a specific impairment in the ability of axonal mitochondria to generate ATP during high demand, and that failed targeting of axonal mitochondria is not solely responsible for defective neurotransmission, but is due to bioenergetic impairment [188].

#### MFF

MFF-knockdown in mouse primary neurons significantly increases axonal mitochondrial size, but interestingly, mitochondria were found to traffic normally, and no difference was observed in the number of presynapses. Moreover, MFF-knockdown has no effect on dendritic mitochondria, even though MFF is expressed in both compartments [12]. The elongated axonal mitochondria are slower to enter the axon, implying that MFF activity within the soma regulates the size of mitochondria entering the axon. No difference in ∆ψm and ATP generation between control and MFF-deficient mitochondria were detected. However, the enlarged mitochondria exhibit greater Ca^2+^ uptake during stimulation, which reduced presynaptic [Ca^2+^], resulting in reduced neurotransmitter release [12].

This study directly links mitochondrial morphology with presynaptic function. Moreover, no differences were detected in dendrites, which raises the possibility that fission is regulated in a compartment-specific manner. Further investigation is required to explore the possibility of differential activity/regulation of fusion/fission proteins between the axon and dendrites.

## Balancing fusion and fission in axons

Axonal mitochondria have the capacity to regulate their size. As mitochondria traffic along axons, they fuse and then subsequently divide, and when they divide, they then undergo fusion with the next mitochondria. This cyclic fusion–fission behaviour occurs the majority (∼85%) of the time and therefore the number of fusion and fission events are equal [55]. Furthermore, in cells overexpressing either Mfn1 or Mfn2, which leads to increased fusion, a concomitant increase in fission has been observed [55]. Likewise, expressing a dominant negative mutant of Mfn2 decreased the number of fusion and fission events. Similar compensatory effects have been observed upon OPA1 ablation [184]. Based on these observations, it appears that smaller mitochondria favour successive fusion events, and larger mitochondria are more likely to undergo consecutive fission events. Thus, axonal mitochondria are capable of ‘self-correcting’ to avoid extremes of mitochondrial size.

DRP1-knockdown, however, reduced the sensitivity of large mitochondria to undergo fission [55], and MFF-knockdown reduces fission events in axons, with no concomitant change in fusion [12], suggesting that axons cannot compensate for the complete impairment of fission, but can for Mfn1/2 disruption, perhaps due to their redundancy. Importantly, the effects on dendritic mitochondria were not reported, so whether a similar self-correcting behaviour occurs in dendrites is yet to be determined.

Interestingly, the mitochondrial morphology upon Marf-knockdown in *Drosophila* can be rescued by the addition of DRP1-knockdown, which also restores mitochondrial function and leads to a partial rescue of mitochondrial density at the NMJ [17]. However, combination of DRP1-knockdown with OPA1 loss did not rescue mitochondrial function, although it did restore mitochondrial morphology and distribution to the NMJ [17]. Thus, mitochondrial morphology and distribution does not necessarily indicate function.

## Mitochondrial dynamics in dendrites

### Role of fusion

#### OPA1

OPA1-knockdown fragments mitochondria in dendrites, which is accompanied by reduced ∆ψm and expression of a number of ETC complex subunits [186]. Fewer PSD-95 positive punctae were observed, indicative of reduced postsynapse formation and/or maintenance, with a concurrent decrease in arborisation of larger dendrites (>60 µm). The distribution of dendritic mitochondria was not significantly different between control and OPA1-knockdown cells, suggesting transport is not impaired. However, the occupancy of mitochondria along dendrites was significantly decreased due to the extensive fragmentation phenotype [186]. OPA1-overexpression in cultured hippocampal neurons does not change the number of mitochondria in the dendrites, but does decrease the size, resulting in a significant decrease in spine density [13]. Thus, both OPA1-knockdown and overexpression decrease mitochondrial occupancy in dendrites, by reducing mitochondrial size, and reduces spine density and dendrite development.

#### Mfn

Purkinje cells in Mfn2^−/−^ mice exhibit severely reduced dendritic process development and cerebellar degeneration [189]. No such phenotype was observed in Mfn1^−/−^ mice. Mitochondria lacking Mfn2 aggregate in the cell body, do not enter the dendrites and display impaired ETC complex activity and loss of mtDNA [189]. Mitochondria in Mfn2-knockout dopaminergic cells exhibit reduced transport and velocity [182], echoing transport defects induced by Miro1 ablation [67]. These findings support the concept that balanced fusion/fission regulates the size and correct distribution within dendrites, and has a significant impact on spine density and dendritic arbour development.

It should be noted, however, that an *in vivo* study of Mfn1-overexpression in pyramidal neurons, which aggregates mitochondria in the soma and significantly reduces mitochondrial trafficking to the dendrites, found an increase in dendritic branch points and length, although the overall arbour was smaller and more compact [18]. Similar results were obtained by overexpressing the TRAK2 mitochondrial binding domain, which disrupts the TRAK-Miro interaction and impairs trafficking. Importantly, this does not affect fusion/fission, therefore suggesting that the aggregated mitochondria can support local, compact, dendritic branch growth. These findings highlight that fusion is required for mitochondrial trafficking into and along dendrites for correct development of the dendritic arbour [18].

### Role of fission

#### DRP1

*In vivo* studies of DRP1-knockout in hippocampal neurons show no gross overall morphological changes to dendritic arbour morphology or spine number [187,188], but dendritic shortening has been observed, despite no impairment in mitochondrial targeting to the dendrite [188]. Overexpression of wild-type DRP1 in cultured hippocampal neurons increases the density of mitochondria along dendrites, with a concomitant increase in spine density [13]. Conversely, expression of a GTPase dead mutant decreased mitochondria along the dendrite and decreased spine density, suggesting that mitochondria are not only required, but also limiting for formation/maintenance of spines [13]. In agreement, DRP1^−/−^ mouse primary neurons have fewer mitochondria within neurites compared to controls due to mitochondrial aggregation within the soma [118], suggesting fission is required in the soma to regulate transport into neurites.

DRP1^−/−^ neurons also developed fewer neurites and synapses [118] and exhibited reduced postsynapse growth, whereas DRP1-overexpression increases postsynapse formation following stimulation [13]. These studies demonstrate that fission is required to control mitochondrial distribution and consequent neurite and synapse development.

A longitudinal study investigating the role of DRP1-knockout in Purkinje cells in mice reported that after 3 months, ∼40% of cells had degenerated, and at 6 months, ∼90% had been lost [190]. Even before cell loss, at 1-2 months, mitochondria appeared swollen in DRP1-depleted cells. Such neuronal atrophy does not occur in hippocampal cells until a much later time *in vivo*, suggesting certain neuronal populations are more susceptible to lack of fission [188]. Cultured Purkinje DRP1-knockout cells have increased ROS and accumulation of mitophagy markers, implying impaired removal of dysfunctional mitochondria. Intriguingly, antioxidant treatment reversed the swollen mitochondrial phenotype and reduced cell death induced by DRP1-depletion [190].

Interestingly, expression of a mitochondrial tethering construct (T20-GFP-GM130C^term^ [191]) *in vivo* in Purkinje cells mimics the aggregation and reduced mitochondrial distribution phenotype seen in fission-deficient cells [30], resulting in a similar dendritic length defect. ATP replacement with creatine rescued the dendritic growth phenotype. These findings indicate that ATP supplied by OXPHOS is primarily responsible for the development and maintenance of dendritic processes, with the turnover of the actin cytoskeleton identified as being a major contributor in developing dendrites [30]. Thus, correct mitochondrial distribution, mediated by fission, is required for neuronal development, synapse formation, and is pivotal in mitochondrial quality control.

#### MFF

MFF-overexpression promotes fission in dendrites [16], and AMPK-mediated phosphorylation of MFF has been reported to promote mitochondrial fragmentation in dendrites [171], but these studies did not report findings regarding axonal mitochondria. This is pertinent because MFF-knockdown was only observed to affect axonal mitochondria [12]. Both studies used *in utero* electroporation to transfect the constructs and visualised mitochondria at similar time points. To address these apparently contradictory observations, it will be necessary to investigate the role of MFF phosphorylation in axons.

#### Mitochondrial fission and synaptic plasticity

As previously mentioned, dendritic mitochondria and fission are implicated in LTP [19]. Chemical induction of LTP (cLTP) in cultured neurons for 15 minutes increases dendritic spine volume by ∼30%, and 1 hour of cLTP increases surface expression of AMPA-type glutamate receptors, which mediate the majority of excitatory neurotransmission. Local dendritic mitochondria undergo extensive fission events within a few minutes of cLTP (termed a ‘fission burst’) [19]. Ca^2+^ influx into the postsynapse correlates with increased fission, and the Ca^2+^-CaMKII-DRP1 signalling axis is responsible for inducing fission in response to cLTP [19].

Disruption of fission inhibited the ‘fission burst’, mitochondrial Ca^2+^ elevations, and subsequently prevented increased spine volume and enhanced AMPAR surface expression, directly implicating dendritic mitochondrial fission in plasticity [19]. Moreover, dendritic mitochondria support spine growth and local protein synthesis during plasticity [16]. Decreasing mitochondrial size, by MFF-overexpression, reduces postsynaptic Ca^2+^ transients and new protein synthesis [16]. How this relates to observations of DRP1-overexpression increasing postsynapse formation following stimulation [13] is yet to be established, but it is clear that altering mitochondrial size by modulating fission has an impact on regulating postsynaptic Ca^2+^ handling, translation and plasticity.

**Figure 5:**
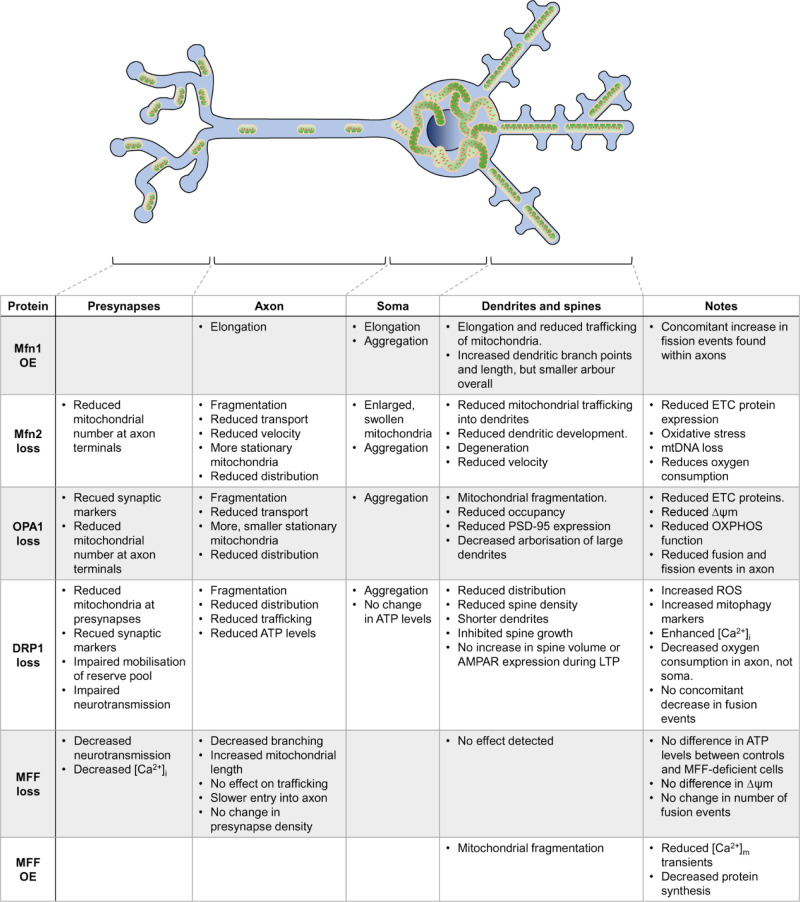
Effects of manipulating the fusion/fission proteins on neuronal mitochondria Summary of the effects on axonal and dendritic mitochondria upon manipulation (knockdown/knockout (loss) or overexpression (OE)) of the fusion and fission machinery. See text for details.

These reports uncover exciting new advances in neuronal mitochondrial biology and directly link dendritic mitochondrial dynamics to the regulation of plasticity. Figure 5 summarises the major findings from manipulations of mitochondrial dynamics outlined here, and the biological outcomes for mitochondrial trafficking, distribution and function.

## Mitochondrial dynamics and disease

Neurodegenerative diseases are characterised by an accumulation of toxic protein, oxidative stress, neuroinflammation, mitochondrial dysfunction and neuronal death, leading to cognitive decline and motor defects. Mitochondrial dysfunction, correlating with increased fragmentation, is a common theme of neurodegeneration, and is thought to be a fundamental underlining early factor in disease progression [192–194].

The most common neurodegenerative diseases are Alzheimer’s and Parkinson’s disease (AD and PD), hallmarks of which include a toxic build-up of amyloid-β and hyper-phosphorylated tau in AD, and α-synuclein in PD [195–197]. These aggregates have direct detrimental effects on mitochondrial function. Amyloid-β localises to mitochondria in the brains of AD patients, is internalised by the mitochondrial import machinery [198] and can impede protein import [199]. Transgenic-mouse strains of amyloid-β and tau pathophysiology exhibit reduced ∆ψm, increased oxidative stress and impaired OXPHOS activity [200]. Likewise, α-synuclein localises with mitochondria *in vivo* and *in vitro*, inhibiting complex I activity [201,202]. Recent findings show α-synuclein interacts with mitochondrial import machinery, disrupting mitochondrial protein import and causing oxidative stress and reducing ∆ψm [203].

Amyotrophic lateral sclerosis (ALS) is characterised by excessive mitochondrial fragmentation and dysfunction, increased ROS and protein aggregation, leading to degeneration of upper and lower motor neurons. Most ALS cases are sporadic, but some have a genetic component, such as those characterised by mutation and aggregation of superoxide dismutase 1 (SOD1) [204]. Huntington's disease (HD) is characterised by aggregation of polyglutamine‐expanded huntingtin (Htt) protein, which impairs OXPHOS function [205], leading to mitochondrial dysfunction and neurodegeneration (reviewed in [206–209]). Mutant Htt has been shown to interact with DRP1, promoting mitochondrial fragmentation and neuronal cell death [210]. *In vivo* studies of brain ischemia have shown that mitochondria are susceptible to dysfunction and mitochondrial respiration is impaired [211–213], and *in vitro* studies of primary neurons confirm mitochondrial dysfunction and morphological changes following ischemic insult [214,215].

Overall, there is compelling and accumulating evidence that perturbations in proteins and pathways that support normal mitochondrial function are a common feature of many neurodegenerative diseases. Thus, better understanding of the mechanisms that cause mitochondrial dysfunction, and devising strategies to combat them, represent major areas of medical research. Below we summarise what is known about how specific mitochondrial proteins, and in particular mutations within them, are implicated in neurodegeneration.

### Impaired fusion in disease

#### Mfn2

Mutations in Mfn2 have been linked to Charcot-Marie-tooth disease type 2A (CMT2A), which involves slow and progressive degeneration of peripheral neurons, mostly affecting the extremities [216]. Mfn2-knockout mice exhibit reduced activity and spontaneous movements, and locomotor-defects, whereas Mfn1-deficient mice show no motor defect [182], agreeing with other reports [189]. Mfn2-deficiency also leads to degeneration and loss of dopaminergic neurons in the midbrain. Investigation of mitochondrial morphology in cultured Mfn2-deficient dopaminergic neurons showed extensive mitochondrial fragmentation, reduced transport and velocity [182]. Mfn proteins can interact with the Miro-TRAK complex, as can CMT2A-related mutants of Mfn2. Mfn2-null neurons, and neurons expressing Mfn2 disease mutants exhibit reduced axonal transport similar to that of Miro-null neurons. Together, this suggests that Mfn2 can regulate the transport machinery [183], but how this is controlled and the significance to disease progression remains to be defined.

#### OPA1

OPA1 mutations are linked to degeneration of the optic nerve, leading to eventual blindness [217,218], with over 100 mutations associated with the disease [219]. Heterozygous knockout mice are viable, but show progressive loss of optic nerve axons [87], with OPA1 loss causing mitochondrial accumulation in the soma, but there are differing reports as to the effect on axonal distribution [17,183–185]. Remarkably, mild overexpression (∼1.5-fold increase) of OPA1 is protective against various insults *in vivo* [220]. For example, following ischemic insult, cell death in the heart and infarct size in the brain were significantly reduced [220]. Indeed, during *in vitro* OGD, OPA1 processing was observed to favour smaller isoforms, indicative of promoting fission [221]. Similarly, OPA1-overexpression can reverse motor and respiratory deficits and increase lifespan in mouse models of mitochondrial disease, with OPA1 being important for regulating cristae structure, stabilising OXPHOS complexes and ameliorating cell death [222]. These studies highlight OPA1 as a potential therapeutic target during mitochondrial disease.

### Impaired fission in disease

#### DRP1

A human DRP1 missense mutation (DRP1^A395D^) has been reported in a new-born [223]; the patient exhibited little spontaneous movement, reduced reflexes, poor visual fixation, and died suddenly aged 37 days. High concentrations of lactate in the blood and spinal fluid were detected, typical of a mitochondrial disease where OXPHOS function is impaired. Fibroblasts taken from the patient showed elongated and interconnected mitochondria, similar to those observed in DRP1^−/−^ cells [223]. Two further reports document other missense mutations in DRP1; a DRP1^R403C^ mutant was found in two unrelated individuals with normal childhood development until the onset, at 4–5 years, of seizures, brain atrophy and developmental delay [224]. A DRP1^G362D^ mutant was identified in an individual who exhibited slow motor skills, epilepsy and seizures within the first years of life, and global developmental delay at 7 years [225]. All these mutations reduce DRP1 oligomerisation and mitochondrial recruitment, and increase mitochondrial elongation [223–226], possibly leading to accumulation within the soma, preventing transport along axons and dendrites and impaired development of synapses.

#### MFF

Autosomal recessive mutations of MFF, producing a truncated protein with no transmembrane domain, cause Leigh-like syndrome in infants. Patients become symptomatic within the first year of life, exhibit developmental delay, microcephaly, progressive development of spasticity, and optic and peripheral neuropathy [227,228]. Using *in vivo* MFF-knockdown in mice, it was shown that layer 2/3 of the cortex exhibited significantly reduced axonal branching and neurotransmitter release [12]. The authors postulate that the reduced synaptic activity leads to the deficit in axonal branching. Indeed, suppression of neurotransmission reduces development of axon branching [229], although further investigation is required to confirm the mechanism of this MFF-dependent axonal branching phenotype, and how this relates to disease.

MFF-null mice display neuromuscular defects and cardiomyopathy, leading to heart failure, and die at an average age of 13 weeks [230]. Cardiomyocytes from MFF-null mice have decreased mtDNA levels and ETC complex activity, and exhibit increased oxidative stress. Remarkably, crossing MFF^−/−^ with Mfn1^−/−^ mice, both lethal on their own, rescued the cardiomyopathy, mitochondrial defects and oxidative stress. A cohort of ten MFF^−/−^/Mfn1^−/−^ mice were followed for 1 year and showed no signs of ill health [230]. Even ablation of one allele of Mfn1 or Mfn2 in the MFF-null mice enhanced life span by ∼60%, although only complete disruption of both Mfn1 alleles restored OXPHOS function and reversed heart dysfunction [230]. Similarly, in *Drosophila* DRP1-knockdown in Marf-deficient flies restores survival rate. However, increased survival was not observed for dual DRP1/OPA1-knockdown, although DRP1-knockdown had the greatest effect at restoring mitochondrial morphology in OPA1-knockdown flies. Thus, the combined DRP1/Marf-knockdown restores mitochondrial function, but DRP1/OPA1 does not [17]. Together, these studies reiterate the importance of balanced fusion and fission for survival, and demonstrate that manipulations of the fusion/fission balance can be protective under disease states. However, it is necessary to be mindful that reversing a morphological phenotype by manipulating mitochondrial dynamics does not necessarily restore function [17].

### Targeting mitochondrial dynamics for therapeutic benefit

#### Targeting mitochondrial proteins – a possible therapeutic strategy?

Recent studies have questioned if manipulating mitochondrial dynamics can rescue mitochondrial dysfunction in disease. Indeed, a growing body of evidence indicates that inhibition of DRP1-mediated fission is neuroprotective in a number of disease models. Mitochondrial division inhibitor mdivi-1, an inhibitor of DRP1, is neuroprotective in a variety of models of ischemic injury, where treatment with mdivi-1 abrogates ROS generation, reduces neuroinflammation and is neuroprotective by blocking apoptosis [231–233]. Mdivi-1 rescues amyloid-β-induced suppression of synaptic transmission, improves cognitive function and reduces neuroinflammation and oxidative stress in a mouse model of AD. DRP1 inhibition also reduced deposition of amyloid-β in the brains of AD mice [234], suggesting that mitochondrial fission exacerbates pathophysiology.

In patient-derived fibroblasts harbouring an ALS mutation in SOD1, inhibiting the Fis1-DRP1 interaction using a small molecule inhibitor (P110) restored mitochondrial function, reduced the mitochondrial morphology phenotype and reduced ROS generation [235]. Furthermore, treatment of NSC-34 cells (motor neuron-like cells) with P110 decreased the recruitment of the mitophagy proteins parkin, p62 and LC3B-ll, and also decreased apoptosis markers. Remarkably, P110 treatment improved behavioural outcomes in an ALS mouse model, rescued mitochondrial structural abnormalities and enhanced lifespan [235]. It is noteworthy that P110 treatment had no effect on control cells, indicating that disrupting this interaction has no adverse effects under physiological conditions [235]. Indeed, P110 is protective against mitochondrial dysfunction and fragmentation following MPP^+^ treatment (mitochondrial complex I inhibitor and neurotoxin that causes Parkinsonism) in SH-SY5Y cells. Furthermore, P110 decreased ROS generation, neurite loss and mitochondrial fragmentation in MPP^+^ treated dopaminergic neuronal cultures [236], indicating that the Fis1–DRP1 interaction is a potential therapeutic target to reduce excessive fission. However, a compound that specifically disrupts the MFF–DRP1 interaction (P259) decreased cognitive and motor functions in a HD mouse model, and also reduced survival rate, while having no effect on wild-type mice [237]. This suggests that inhibition of DRP1, or at least its interaction with certain receptors, may be deleterious in some disease states.

Overall, an emerging concept is that suppression of DRP1 activity confers protection in a range of disease states. However, there is evidence suggesting that the biological outcomes of the Fis1–DRP1 and MFF–DRP1 interactions are different under normal and disease states. Further investigation is necessary to dissect the roles of individual DRP1–receptor interactions, under physiological and pathophysiological conditions, and the potential for targeted therapeutic benefit.

#### Targeting PTMs of mitochondrial proteins – a possible therapeutic strategy?

PTMs of mitochondrial dynamics proteins have been shown to be altered under disease states, leading to mitochondrial dysfunction. For instance, pDRP1^S616^ is enhanced in HD, and inhibition of the MAPK-pDRP1^S616^ pathway corrects mitochondrial fragmentation, restores ∆ψm and reduces ROS [238]. S-Nitrosylation of DRP1 is also enhanced in HD, which causes hyperactivation of DRP1 by increasing the level of pDRP1^S616^. A non-nitrosylatable mutant (DRP1^C644A^) is sufficient to block mitochondrial fragmentation and dendritic spine loss [166]. Similar results were obtained in a cell-based system of AD, whereby expression of DRP1^C644A^ prevents dendritic spine loss and reduces cell death [165]. Treatment of neurons with amyloid-β causes a CDK5-dependent increase in pDRP1^S616^ levels, and CDK5-knockdown or chemical inhibition of CDK5 activity attenuates amyloid-β-induced mitochondrial fission and apoptosis [239]. Moreover, amyloid-β causes elevations in Ca^2+^, activating the CaMKII-pDRP1^S616^ pathway. Blocking this pathway prevented DRP1 recruitment and reduced amyloid-β induced neurotoxicity, mitochondrial dysfunction and apoptosis [240].

Together, these studies demonstrate that although DRP1-mediated fission is involved in plasticity [19], it can lead to neurodegeneration when overactivated in disease states. Furthermore, they highlight the possibility of targeting DRP1 phosphorylation for therapeutic benefit in a number of diseases.

Calcineurin-mediated dephosphorylation of pDRP1^S637^ has been implicated in MOM recruitment and neuronal cell death in *in vitro* [241] and *in vivo* [242] models of ischemia. Inhibition of the calcineurin-DRP1 interaction [241], or chemical inhibition of calcineurin, is protective against ischemia-induced neuronal cell death [242]. MEK/ERK-mediated phosphorylation of Mfn1^T562^ has been implicated in apoptosis, and expressing a phospho-null mutant (Mfn1^T562A^) is protective against OGD-induced neuronal cell death [105]. SUMO2/3-ylation of DRP1 in neurons partitions DRP1 in the cytosol during OGD [162], by reducing DRP1 binding to MFF [163]. Upon reperfusion, DRP1 is deSUMOylated, initiating translocation to the mitochondria and inducing fragmentation and cell death [162]. These studies demonstrate that individual proteins and specific modifications can be targeted to confer neuroprotection. How they relate to one another, and under what conditions, requires further investigation.

The role of, and mutations in, the PINK1/parkin pathway in mitochondrial quality control and PD have been reviewed extensively [140,243,244]. Recent research indicates that lack of parkin or PINK1 in mice exacerbates inflammation, leading to motor defects and loss of dopaminergic neurons [245]. Increased inflammatory markers were also detected in the serum of human PD patients. Blocking the STING pathway (a central regulator of the inflammatory response to cytosolic DNA) reduces motor defects and neuronal loss in PD-mutant mice, indicating that the PINK1/parkin pathway prevents inflammation by clearing damaged mitochondria [245], inferring that inflammation may be a primary factor in neuronal cell loss. However, this model has recently been challenged by a study in *Drosophila*, which found no role of STING in PD [246]. As previously mentioned, Mfn1/2 and MFF are targets of parkin-mediated ubiquitination and required for mitophagy [107–109,174], but interestingly, parkin conjugates atypical poly-ubiquitin chains to Mfn1 [113] and MFF [175]. Whether these modifications have a role in this pathway, and whether they could be targeted therapeutically, offers an exciting new area of research.

## Conclusion and perspectives

Remarkable progress has been achieved towards understanding the regulation of mitochondrial dynamics and the roles it plays in neurons. Recent advances have begun to elucidate why mitochondria exhibit different morphologies in the axon and dendrites, and have revealed how mitochondrial dynamics are regulated by PTMs and the complex interplay between these modifications. Here we have outlined the vital roles of neuronal mitochondria in modulating Ca^2+^, generating ATP during activity, regulating neurotransmission and influencing plasticity. Further, we outline that balanced fusion/fission is important for transport, and that impaired mitochondrial dynamics leads to impaired synapse formation, neurite integrity and degeneration.

However, despite this progress, much remains to be determined. For example, how is the differential regulation of mitochondrial fusion/fission proteins between the axon and dendrite coordinated? Why are mitochondria more dynamic in axons than in dendrites, and is this related to different interactions with the cytoskeleton? Axons have a ‘self-correcting’ ability to avoid extremes of size, but how is this regulated, and is there such a mechanism in place in dendrites? Are the larger mitochondria in the dendrites connected with a greater Ca^2+^ buffering capacity? Possible explanations for these questions have been presented here, but a greater understanding of the modulation of mitochondrial dynamics, their compartment-specific regulation and how they respond during plasticity will no doubt advance our appreciation of the morphological and functional differences of neuronal mitochondria.

As set out, mitochondrial dysfunction is a common fundamental characteristic of neurodegenerative disorders, and dysregulated PTMs of the fusion/fission machinery have been implicated in promoting pathophysiological mitochondrial function. Greater understanding of the processes of mitochondrial dynamics, and how they are dysregulated in disease states, offers a promising target for therapeutic benefit.

## Abbreviations

AD: Alzheimer’s disease
ADP: adenosine diphosphate
ALS: amyotrophic lateral sclerosis
AMPAR: α-amino-3-hydroxy-5-methyl-4-isoxazolepropionic acid receptor
AMPK: AMP-activated protein kinase
ATP: adenosine triphosphate
CaMK: Ca^2^+/calmodulin-dependent protein kinase
CCCP: carbonyl cyanide m-chlorophenyl hydrazine
CDK: cyclin-dependent kinase
cLTP: chemically induced long-term potentiation
CMT2A: Charcot-Marie-tooth disease type 2A
DRP1: dynamin related protein 1
EPSC: excitatory postsynaptic current
ER: endoplasmic reticulum
ERK: extracellular-signal-regulated kinase
ETC: electron transport chain
Fis1: mitochondrial fission protein 1
GTP: guanosine triphosphate
HD: Huntington's disease
HDAC: histone deacetylase
Htt: polyglutamine-expanded huntingtin
IMS: intermembrane space
LTP: long-term potentiation
MARCH5: membrane associated RING-finger 5
MCU: mitochondrial Ca^2+^ uniporter
Mdivi-1: mitochondrial division inhibitor
MEK: mitogen-activated protein kinase kinase
MFF: mitochondrial fission factor
Mfn: mitofusin
MiD: mitochondrial dynamics protein
MIM: mitochondrial inner membrane
∆ψm: mitochondrial membrane potential
MOM: mitochondrial outer membrane
NMJ: neuromuscular junction
OGD: oxygen-glucose deprivation
OPA1: optic atrophy protein 1
OXPHOS: oxidative phosphorylation
PD: Parkinson’s disease
PINK1: PTEN-induced kinase 1
PP2A: protein phosphatase 2
PSD-95: postsynaptic density protein 95
ROS: reactive oxygen species
SENP: sentrin-specific protease
SIMH: stress-induced mitochondrial hyperfusion
SOD1: superoxide dismutase 1
STING: stimulator of interferon genes
SUMO: small ubiquitin-like modifier
TCA: tricarboxylic acid
TRAK: trafficking kinesin protein
VDAC: voltage-dependent anion-selective channel
